# The Heritability of Migration Behaviors in a Wide‐Ranging Ungulate

**DOI:** 10.1002/ece3.74024

**Published:** 2026-07-13

**Authors:** Maegwin Bonar, Eric Wootton, Charles R. Anderson, George Wittemyer, Aaron B. A. Shafer, Joseph M. Northrup

**Affiliations:** ^1^ Environmental & Life Sciences Graduate Program Trent University Peterborough Ontario Canada; ^2^ Molecular Biology & Biochemistry Undergraduate Program Trent University Peterborough Ontario Canada; ^3^ Mammals Research Section Colorado Parks and Wildlife Fort Collins Colorado USA; ^4^ Department of Fish, Wildlife and Conservation Biology Colorado State University Fort Collins Colorado USA; ^5^ Wildlife Research and Monitoring Section Ontario Ministry of Natural Resources Peterborough Ontario Canada

**Keywords:** animal model, genomic relatedness matrix, heritability, migration

## Abstract

Migration behavior is thought to be declining globally in the face of rapid human‐mediated environmental change. Although many species exhibit individual plasticity in their migratory behavior, not all species demonstrate the level of plasticity necessary to adjust to novel conditions. Selection on heritable behaviors might therefore play an important role in the maintenance of migratory phenotypes for some species. Using GPS and genomic data from 143 individuals (151 animal‐years) in a pedigree‐free quantitative genetic approach, we estimated heritability, repeatability, and sources of environmental variation for migration traits in migrating mule deer ( *Odocoileus hemionus* ). We found low heritability and low repeatability for broad patterns of migration timing, and moderate heritability and repeatability for distance, duration, and movement rate along the migratory route. Our findings suggest that wild mule deer populations have the potential to respond to selection pressure generated by global environmental changes through microevolutionary changes in migration behaviors.

## Graphical abstract

Despite our understanding that variation in migration behavior has both genetic and environmental components, studies quantifying the sources of genetic variation contributing to migration phenotypes are lacking. Our study provides, to our knowledge, the first empirical evidence of heritability in migration behavior in ungulates. These results have implications for the evolution and maintenance of migration behaviors in natural populations.

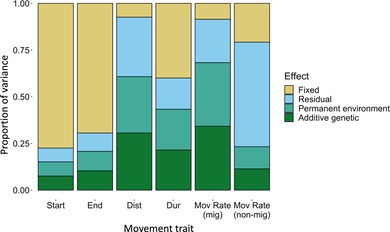

## 1 Introduction

Migration behavior is critical for the reproduction and survival of a wide variety of taxa, yet populations have experienced global declines in migrations in the face of rapid human‐mediated environmental change (Aikens et al. 2017; Harris et al. 2009; Tucker et al. 2018; Wilcove and Wikelski 2008). Although many migrating species experiencing rapidly changing environments can adapt to novel conditions through behavioral plasticity (Eggeman et al. 2016; Xu et al. 2021), not all species demonstrate high levels of individual plasticity (Sawyer et al. 2019). In this case, selection on heritable behavioral components might play a stronger role in the maintenance of migratory phenotypes than previously thought. Inference on the selection, evolution and maintenance of migration behavior is often based on phenotype alone with the assumption that phenotypic variation is an adequate predictor of underlying genetic differences (i.e., the phenotypic gambit; Grafen 1984). Whether the phenotypic gambit holds or not, is unclear for many taxa yet critically important as the proportion of phenotypic variation of migratory traits explained by genetic differences could provide insight into the adaptive potential of a migrating species facing rapid global environmental change.

The proportion of phenotypic variance explained by additive genetic variance (i.e., narrow‐sense heritability) is a key determinant of the rate of adaptive evolution. Quantifying the genetic variance underpinning phenotypic variation can be used to make inferences about the inheritance and evolutionary potential of those traits without explicit knowledge of the genetic loci involved. However, calculating the additive genetic variance in natural systems is challenging, because such metrics rely on knowing both the trait values for individuals and their relatedness to one another. Classically, this is accomplished with multigenerational pedigrees, however sequencing technologies have made the estimation of genomic relatedness matrices (GRMs) more frequent (Speed and Balding 2015). GRMs are now being used to estimate heritability in natural populations, broadening our understanding of the adaptive potential of many species (Stanton‐Geddes et al. 2013). This approach has been used to estimate heritability in many traits such as body size (Bérénos et al. 2014; Jamieson et al. 2020; Perrier et al. 2018), stress response (Gervais et al. 2020; Oikonomou et al. 2022), aggression and docility (Gervais et al. 2020; Taylor et al. 2012), and more recently movement and space‐use behaviors (Gervais et al. 2020). However, this approach has yet to be applied to quantify the genetic variance underpinning movement behaviors in migrating populations.

In this study, we used a pedigree‐free quantitative genetic approach to quantify the proportion of phenotypic variance explained by additive genetic effects in spring migration behaviors in migrating mule deer ( *Odocoileus hemionus* ). In the spring, migration behavior is linked to reproductive success as female mule deer migrate from their winter range to give birth on their summer range (Lendrum et al. 2014). Mule deer exhibit variation in components of their migration behavior such as migration distance (Sawyer et al. 2019), and timing (Northrup et al. 2014), and this variation could lead to variation in fitness. In the case of migration timing, mule deer typically migrate to match changes in resource availability, and optimize migratory timing relative to both plant productivity and weather on their summer range (e.g., Lendrum et al. 2013). However, the fitness consequences of a mismatch in timing with the flush of high‐quality vegetation in spring (i.e., spring green‐up) may vary (Aikens et al. 2017; Rivrud et al. 2018). For example, arriving ahead of spring green‐up and facing early spring storms could result in mortality, but in a milder year would mean first access to the best areas for giving birth. Migrating longer distances can increase exposure to anthropogenic mortality factors such as highways or fences, while migrating shorter distances can increase vulnerability to harvest from human hunters (Sawyer et al. 2016). Migrating at a faster movement rate could decrease exposure to predation along the route and lower mortality risk, but this would come at a cost of physical condition at an already taxing life‐history stage (Ydenberg and Hope 2019). Partial migration is common in mule deer populations (Nicholson et al. 1997), however mule deer exhibit little to no individual variation in migratory plasticity in terms of whether or where to migrate (Sawyer et al. 2019). Consequently, adaptation to a rapidly changing environment might require microevolutionary change, for which a degree of heritability for migratory variation is required.

Broadly, behavior is often assumed to be highly plastic, suggesting that the magnitude of additive genetic variance should be small relative to the effect of environmental variation (Dingemanse et al. 2010; Mery and Burns 2010). Accordingly, we would expect the heritability of migration traits to be low compared to other traits such as morphological traits. A recent meta‐analysis on the heritability of behavioral traits across vertebrate and invertebrate taxa by Dochtermann et al. (2019) reported the range of heritability of behavioral traits is 0.24–0.31. Although Dochtermann et al. (2019) grouped migration and dispersal together into a single behavioral category in their models, they estimated moderate heritability of these traits (*h* ^2^ = 0.46; 95% CI: 0.33–0.57). Given our current understanding of the genetic and environmental drivers of migration (Abraham et al. 2022; Bonar et al. 2023; Sawyer et al. 2016), we predicted a larger proportion of phenotypic variation in migration behavior to be explained by environmental effects. We predicted additive genetic effects (i.e., heritability) to explain a moderate proportion of phenotypic variance. We predicted low heritability for migration timing, as variation in migration timing across ungulates is known to be heavily influenced by plant phenology (Abraham et al. 2022) and environmental effects. We predicted moderate heritability for migration distance, duration, and movement rate traits as movements along the route are subject to greater individual variation. We predicted high levels of repeatability for all migration traits in mule deer given the low levels of plasticity in migratory behavior previously reported (Laforge et al. 2025; Sawyer et al. 2019).

## 2 Materials and Methods

### 2.1 Study Site

**FIGURE 1:**
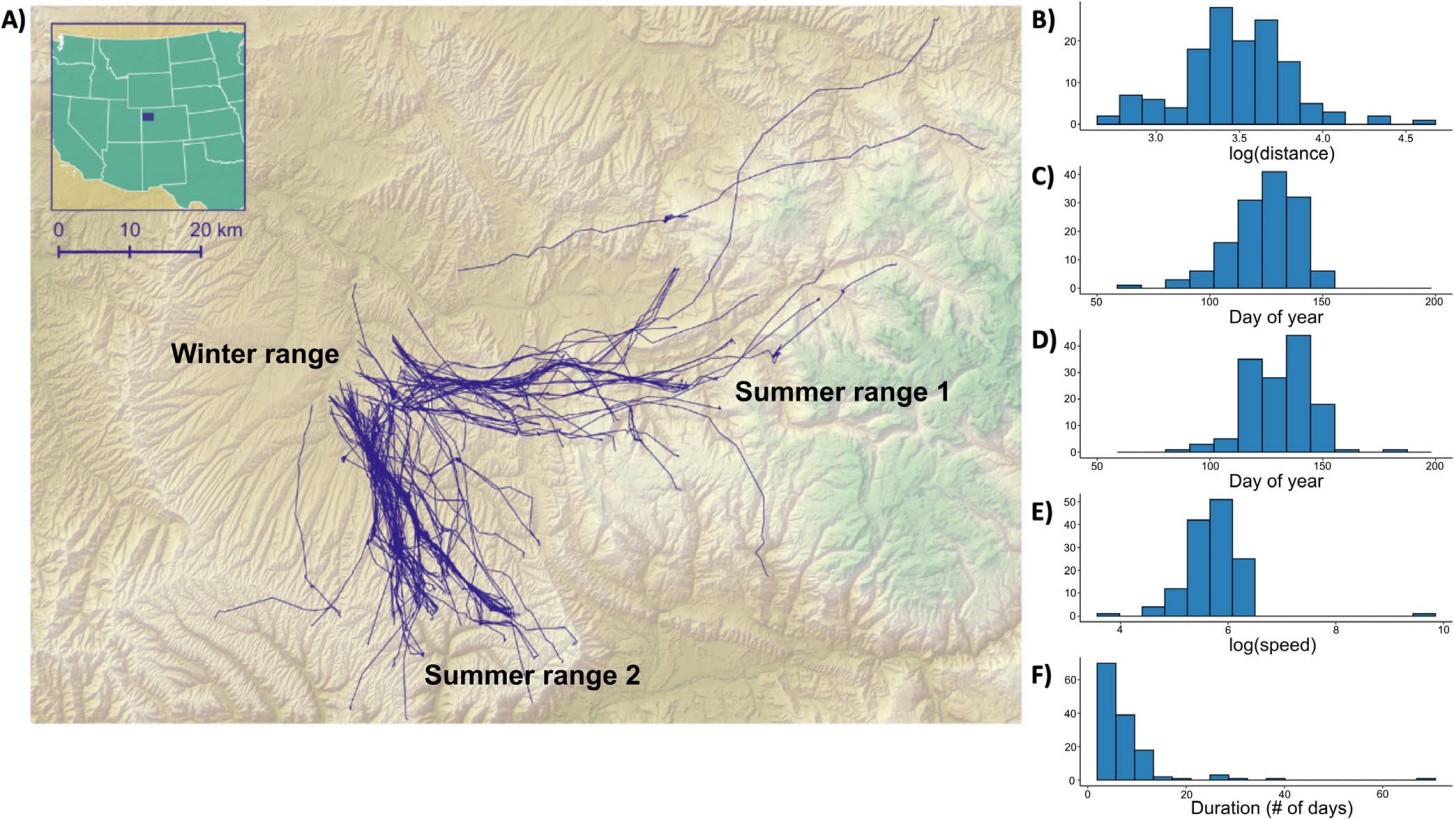
(A) Adult female mule deer spring migration routes for 151‐animal years in the Piceance Basin, Colorado, USA. Mule deer in this area typically occupy a single winter range and migrate to two different summer ranges. Frequency distributions of migratory traits for (B) log‐transformed Euclidean migration distance; (C) migration start day; (D) migration end day; (E) log‐transformed average movement rate along the route; and (F) duration of migration.

This study took place in the Piceance Basin of northwestern Colorado, USA. The climate is characterized by cold winters (range = −37.2°C–22.8°C) and warm dry summers (range: −2.2°C–35.6°C) with monsoonal precipitation in the late summer (Northrup et al. 2021). The area is topographically variable with the dominant vegetation consisting of big sagebrush ( *Artemisia tridentata* ) and a pinyon pine ( *Pinus edulis* ) Utah juniper ( *Juniperus osteosperma* ) shrubland complex. Other dominant shrubs include Utah serviceberry (*Amalenchier utahensis*), mountain mahogany ( *Cercocarpus montanus* ), bitterbrush ( *Purshia tridentata* ), and mountain snowberry ( *Symphoricarpos oreophilus* ). This area is popular for hunting during the fall with an annual average of 511 deer harvested in the wildlife management unit (Game Management Unit 22), which encompassed the entire study area (Northrup et al. 2021). Mule deer in this area typically occupy their winter range between October and April of each year (Lendrum et al. 2014; Northrup et al. 2014) and migrate to two different summer ranges (Lendrum et al. 2014) located south or east of the winter range (Figure 1). Elevation on the winter study area ranges from 1675 to 2285 m and from 2000 to 2800 m on the summer study areas. Previous work in this site indicates there is no genetic subdivision within the population based on migration to the two summer ranges (Bonar et al. 2022; Northrup et al. 2014).

### 2.2 Individual Sampling and Phenotyping

Adult (> 1 year old) female mule deer were captured using helicopter net gunning and fitted with store‐on‐board GPS radio collars (Advanced Telemetry Systems, Isanti, MN, USA) between 2012 and 2016. Deer were spotted visually by the helicopter capture crew and captured using a net gun. Deer were then blindfolded, hobbled, and administered 0.5 mg/kg of Midazolam and 0.25 mg/kg of Azaperone intramuscularly to alleviate capture‐related stress (dose of both drugs based on an average weight of 75 kg). Deer were transported to a central processing site typically within 2 km of the capture site, where they were weighed, measured for chest girth and hind foot length, and blood samples were collected for genetic analysis. We estimated their age in years using tooth replacement and wear (Hamlin et al. 2020; Robinette et al. 1957; Severinghaus 1949). We also obtained a body condition score by palpating the rump and measured the thickness of subcutaneous rump fat and the depth of the longissimus dorsi muscle using ultrasound (Cook et al. 2001, 2007, 2010; Stephenson et al. 1998, 2002). We used the body condition score and ultrasound measurements to estimate the percent ingesta‐free body fat of each deer in March of the year of capture (Cook et al. 2007, 2010). During late‐winter captures, we assessed pregnancy using ultrasound and for does for which we did not detect a fetus, we confirmed pregnancy status using pregnancy‐specific protein B from blood samples. Deer were released at the processing site immediately following blood sample collection and GPS collar attachment. All procedures were approved by the Colorado State University (protocol ID: 10‐2350A) and Colorado Parks and Wildlife (protocol ID: 15‐2008) Animal Care and Use Committees.

We input GPS locations into Migration‐Mapper v3.0 (Merkle et al. 2022) and followed Modules 1–3 as described in the Migration‐Mapper user guide https://migrationinitiative.org/projects/migration‐mapper/migration‐mapper‐user‐guide/ where we removed erroneous locations (e.g., unreasonable speeds or turning angles; Merkle et al. 2022), and then delineated migration routes visually for each individual using graphs of the individual's net‐squared displacement. Six individuals were collared for more than 1 year, so we determined spring migrations for every year in which data were available. After migration routes had been determined, we recorded the start and end of spring migration in day‐of‐year based on the relocation date at the start and end of each migration route. We calculated the duration of the migration in days and calculated Euclidean distance from the first GPS location of the migration route to the last GPS point (Joly et al. 2019). The GPS radio collars were programmed with a 5‐h, 1‐h, or 30‐min relocation schedule depending on the individual and year sampled. To account for the three different relocation schedules, we rarefied the finer scale data to match the resolution of the 5‐h data for all subsequent analyses. Movement rate in m/h was calculated per single step length (i.e., the distance traveled between two successive GPS locations) and then averaged across all step lengths for the entire route. Habitat characteristics (i.e., vegetation type) occurring along the migratory paths of mule deer were similar across the study areas (Lendrum et al. 2013), however individuals that migrated east–west on average traveled greater distances than individuals migrating north–south.

### 2.3 DNA Extraction and Library Preparation for RAD Sequencing

We extracted DNA from blood samples for all radio collared individuals using the DNeasy Blood and Tissue Kit (Qiagen Inc., Valencia, CA, USA), following the manufacturer's protocol. We generated restriction site‐associated DNA sequencing (RADseq) libraries using an adapted protocol from Parchman et al. (2012) and Peterson et al. (2012). Samples were incubated, digested overnight, and heat‐inactivated in 96‐well plates (see Haworth et al. 2021) for reaction conditions and primer details. Each 96‐well plate had UltraPure distilled water (Invitrogen, 1,897,011) as negative controls. Restriction digested DNA was combined with 7 μL of ligation mixture and 3 μL of one of the 24 available Sbfl adapters (1.0 μM), and adapters were ligated at 16°C for 3 h. We purified DNA fragments of artifacts following manufacturer protocol for AMPure XP beads (A63880; Beckman Coulter). Adapter‐ligated fragments were amplified in four separate 10 μL reactions that incorporated barcodes. We pooled and purified samples following manufacturer protocol for QIAquick PCR Purification kit (28,106; Qiagen) for a final elution to 42 μL. We performed size selection between 450 bp to 700 bp on 80 μL purified libraries and performed gel purification following manufacturer protocol for QIAquick Gel Extraction kit (28,706; Qiagen) for a final elution to 60 μL. We characterized the pooled and purified final libraries with on a TapeStation using the D1000 kit (5067–5582; Agilent). The libraries were sequenced at The Centre for Applied Genomics (TCAG) in The Hospital for Sick Children (SickKids), Toronto, Canada on an Illumina HiSeq 2500 to produce 2 × 126 base pair paired‐end reads.

### 2.4 Bioinformatic Pipeline for RADseq Data

Fastq files were demultiplexed using *process_radtags* within the Stacks v2.3 module (Rochette et al. 2019). Parameters within *process_radtags* included the removal of any read with an uncalled base and the discarding of reads with low‐quality scores. The demultiplexed sample files were aligned against the white‐tailed deer genome ( *Odocoileus virginianus* ) (NCBI PRJNA420098; Accession No. JAAVWD000000000) that was recently annotated (Anderson et al. 2022). Mule deer and white‐tailed deer can hybridize (Stubblefield et al. 1986), so we opted to use the long‐read‐based draft genome of white‐tailed deer. We note that the mule deer genome available at the time of analysis was simply a consensus sequence from reads mapped to earlier versions of the white‐tailed reference (Russell et al. 2019). Raw reads had a 98.6% mapping rate. Mapped reads were sorted and indexed using SAMtools (Li et al. 2009). We then ran the *gstacks* and *populations* program within STACKs, retaining loci found in at least 80% of samples (*r* = 0.80), with a minor allele frequency of 1% (min_maf = 0.01), and heterozygosity upper bound of 0.8 (max_het = 0.8) that produced a variant call format (VCF) file. We also only retained one single nucleotide polymorphism (SNP) per locus (—write_single_snp) to meet the assumptions of linkage equilibrium in subsequent analyses.

The VCF file was converted into a binary fileset using PLINK v.19 (Purcell et al. 2007) and the VCF file was filtered to only include individuals with < 10% missing data. Using VCFtools (Danecek et al. 2011), we determined F_IS_ and observed and estimated homozygosity and removed outlier individuals with *F*_IS_ < −0.1 on the basis of excess heterozygosity likely from contamination (Cericola et al. 2018; Simeone et al. 2011), which was visualized with a principal component analysis (Figures S1, S2) using PLINK and R v.4.2.1 (R Core Team 2022). The VCF file was converted into a binary fileset using PLINK and that was used to generate the classic genomic relatedness matrix (GRM) in GCTA v1.92.4 (Yang et al. 2011). We examined the diagonal values of the GRM to identify potential problematic samples with inflated self‐relatedness values and used this to further inform filtering.

After applying quality filtering on the RADseq data, all 143 individuals were retained in the unfiltered GRM. The GRM included 10,097 SNPs and 20,306 pairwise relatedness coefficients, of which 109 pairs (116 animal‐years) had a relatedness coefficient higher than 0.1 (Figure S3, Table S1). Highly related individuals were removed at two levels (*r* > 0.10 and *r* > 0.05) to reduce heterogeneity in the matrix. The reason for this exclusion was to estimate the genetic variation captured by all SNPs from the RADseq data, similar to the approach taken in genome‐wide association studies (GWASs). Including a small number of closely related individuals within a sample of mostly unrelated individuals could result in a biased estimate of genetic variance that is driven by the phenotypic correlations of these closely related pairs (Yang et al. 2011). The models that used the GRM with a relatedness cutoff of 0.1 included 109 individuals, had a mean diagonal of 1.02, and the total variance of the relatedness coefficient of the off‐diagonal was 0.0001. We also performed all analyses with an unfiltered GRM and a GRM with a relatedness cutoff of 0.05. The results presented herein are for the models that used the GRM with a relatedness cutoff of 0.1. We also performed all analyses with an unfiltered GRM and a GRM with a relatedness cutoff of 0.05. The results of those models can be found in the Table S2.

### 2.5 Quantitative Analyses

We used the animal model approach to partition the phenotypic variance of migration (*V*_P_) into additive genetic variance (*V*_A_), permanent environmental effects (*V*_PE_), and residual variance (*V*_R_) such that *V*_P_ = *V*_A_ + *V*_PE_ + *V*_R_ conditional on any fixed effects. Table 1 summarizes the fixed and random effects for each animal model. We generated an animal model to partition phenotypic variation for the following migration traits: start day of migration, end day of migration, distance traveled, duration traveled, and movement rate along the route. To differentiate the migratory movement rate with that of non‐migratory movement rate, we generated an animal model of non‐migratory movement rate on the winter range using daily average movement rate for just the month of March. We included age and migration year as a fixed effect in all the migration models; summer range was included as a fixed effect for start day, end day, distance, and duration models to account for the variation in distance and elevation of the two summer ranges; log‐transformed distance of migration route was included in the movement rate (both migratory and non‐migratory) and duration models. To test for potential correlation between movement rate and hindleg length, we fit a bivariate animal model (de Villemereuil 2018) with age, year of migration, and log‐transformed distance of migration route as fixed effects. Individual identity was included as a random effect in all the migration models to account for permanent environmental effects (de Villemereuil 2018).

**TABLE 1:** Summary of fixed and random effects for each animal model partitioning the phenotypic variation in migration traits in adult female mule deer in the Piceance Basin of northwestern Colorado, USA.

Model	Response variable	Fixed effects	Random effects	Model distribution
1	Start day	Age + summer range + year	Relatedness (GRM) + identity	Poisson
2	End day	Age + summer range + year	Relatedness (GRM) + identity	Poisson
3	Distance	Age + summer range + year	Relatedness (GRM) + identity	Normal
4	Duration	Age + summer range + log(distance) + year	Relatedness (GRM) + identity	Poisson
5	Movement rate (migration)	Age + log(distance) + year	Relatedness (GRM) + identity	Normal
6	Movement rate (non‐migratory)	Age + log(distance) + year	Relatedness (GRM) + identity	Normal
7	Movement rate (migration):Hindleg length	Age + log(distance) + year	Relatedness (GRM) + identity	Normal

*Note:* All animal models require a measure of pairwise relatedness as a random effect; our measure of relatedness is determined using genomic relatedness matrices (GRMs). We log‐transformed response variables with continuous distributions to meet assumptions of homoscedasticity and normality of residuals and used a Poisson distribution for the discrete response variables.

We included the GRMs in the models as a random effect as our measure of pairwise relatedness. The unfiltered GRM was nonpositive definite but could nevertheless be implemented in an animal model by bending the matrix (Meyer 2019). We also generated animal models to partition the phenotypic variance of body size traits. This allowed us to compare our estimates to those of heritable traits previously described for ungulates (Jamieson et al. 2020; Williams et al. 1994; Wilson et al. 2005). We estimated heritability of body size traits (chest girth, hindleg length, ingesta‐free body fat, and weight), using the same approach as the migration traits. Model details for body size traits can be found in Table S3. We included age, month of capture (December or March), and year as fixed effects in all body size models as body size may differ between life‐history stages and time of year (Monteith et al. 2011). There was little variation in pregnancy rates as 95% of the deer captured in March were pregnant at the time.

We fitted the animal models using a Markov Chain Monte Carlo for generalized linear mixed models with the MCMCglmm package in R (Hadfield 2010) with priors *V* = 1, nu = 0.002. We ran the algorithm 4 times for each model, resulting in 4 chains, and thinned the chains at an interval of 100. We used a burn‐in period of 10,000 iterations and a total of 500,000 iterations per chain to estimate the posterior distribution. We decided to log‐transform traits with continuous distributions to ensure proper support with model assumptions. We ran the start day, end day, and duration models using a Poisson distribution (O'Hara and Kotze 2010). Narrow sense heritability conditional on fixed effects was estimated as *h* ^2^ = *V*_A_/(*V*_A_ + *V*_PE_ + *V*_R_ + *V*_fixed_) and the permanent environmental effect conditional on fixed effects as pe^2^ = *V*_PE_/(*V*_A_ + *V*_PE_ + *V*_R_ + *V*_fixed_). We measured individual repeatability as the ratio of among‐individual variance (genetic and nongenetic) over the total phenotypic variance ind^2^ = *V*_A_ + *V*_PE_/(*V*_A_ + *V*_PE_ + *V*_R_ + *V*_fixed_) (Nakagawa and Schielzeth 2010). Finally, we estimated evolvability for each migration trait (*I*_A_) (Hansen et al. 2011; Houle 1992). Evolvability defined as the mean standardized proportion of additive genetic variance was calculated as *I*_A_ = *V*_A_/*m* ^2^, where m is the trait mean (Hansen et al. 2011). For the models using the Poisson distribution, we report the heritability on both the latent scale and observed data scale, which were determined using the R package QCglmm (de Villemereuil et al. 2016).

## 3 Results

We captured a total of 143 adult female mule deer with sufficient GPS data to determine the migration routes. The number of spring migrations per individual ranged from 1–2 (151 animal‐years total). Frequency plots of migration traits are illustrated in Figure 1. The average start day of spring migration was May 3 (range March 8–June 1) and the average end day was May 10 (range April 1–July 6). The average duration of migration was 7 days (range 2–67). The average distance of migrations to the eastern summer range was 39.8 km (range 14.2–94.9 km) and the average distance to the southern summer range was 31.1 km (range 14.7 km–58.2 km). The average movement rate during migration for individuals was 327.1 m/h (range 41.3–1468.3 m/h), while the average non‐migratory movement rate was 71.2 m/h (range 29.8–175 m/h; Figure S4, Table S4).

**FIGURE 2:**
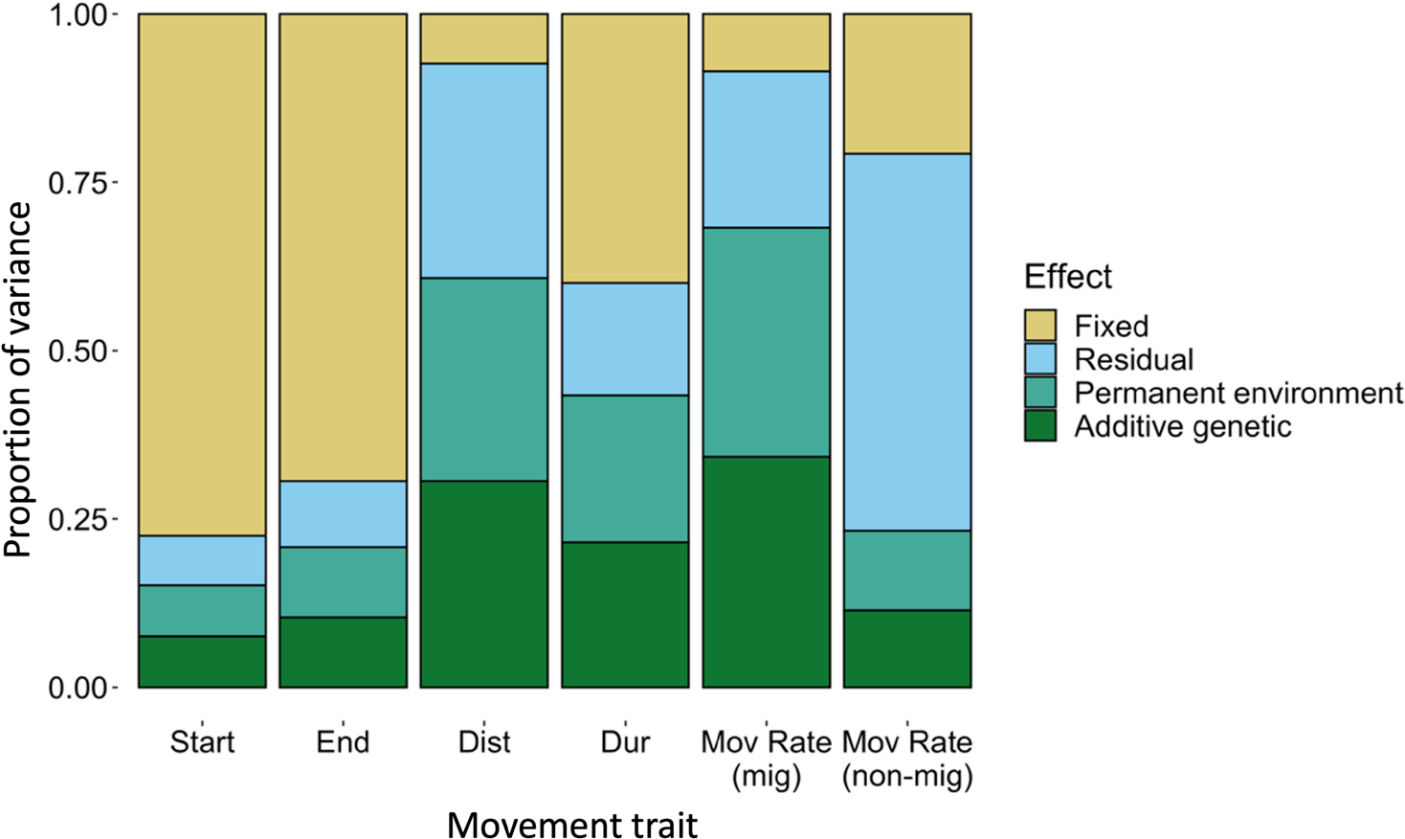
Variance partitioning of spring migration behavioral traits in adult female mule deer (Start, start day of migration; End, end day of migration; Dist, distance migrated; Dur, migration duration; Mov Rate (mig), migratory movement rate; Move Rate (non‐mig), non‐migratory movement rate). Proportion of phenotypic variance of traits (*V*_P_) explained by additive genetic variance (*V*_A_), permanent environmental effects (*V*_PE_), and residual variance (*V*_R_) such that *V*_P_ = *V*_A_ + *V*_PE_ + *V*_R_ conditional on any fixed effects. See Table 1 for a summary of the fixed and random effects for each animal model.

Heritability for start day and end day of migration was low, with observed state values of 0.08 (95% CI 0.03–0.19) for start day and 0.09 (95% CI 0.03–0.22) for end day. Variation in migration start and end day was explained primarily by fixed effects (Figure 2, Table 1, Figure S5, Table S4). Heritability of movement behaviors along the migration route ranged considerably, with migration duration having lower heritability (observed state: 0.02 (95% CI < 0.01–0.12)), whereas average movement rate and distance traveled were moderately heritable at 0.34 (95% CI < 0.01–0.87) and 0.31 (95% CI 0.01–0.79) respectively. Non‐migratory movement rate had low heritability at 0.11 (< 0.01–0.44).

Variance explained by permanent environmental effects was close to the proportion explained by additive genetic variance (Figure 2, Table 2), and both distance traveled and migration movement rate were highly repeatable between individuals 0.61 (95% CI 0.13–0.86) and 0.68 (95% CI 0.05–0.92) respectively. Migration duration was moderately repeatable at 0.43 (95% CI 0.02–0.72). Evolvabilities were low, the evolvability of migratory movement rate was 0.45% (95% CI < 0.01%–1.32%), and the evolvability of migration distance was 0.33% (95% CI 0.01%–0.93%). The bivariate model of migratory movement rate and hindleg length revealed no additive genetic correlation between the two traits (rescaled genetic covariance rG posterior mean 0.01 (95% CI −0.61–0.59), and the estimate of heritability from the bivariate model for migratory movement rate was 0.35 (95% CI < 0.01–0.92)), similar to that of the univariate model estimate (Table S7).

**TABLE 2:** Variance component estimates (mean posterior and 95% CI) and their associated ratios for migration traits in adult female mule deer in the Piceance Basin of northwestern Colorado, USA.

	Start day	End day	Distance	Duration	Migratory movement rate	Non‐migratory movement rate
*h* ^2^ ^a^	0.08 (0.03–0.19)	0.09 (0.03–0.22)	0.31 (0.01–0.79)	0.02 (< 0.01–0.12)	0.34 (< 0.01–0.87)	0.11 (< 0.01–0.44)
pe^2^	0.08 (0.02–0.18)	0.10 (0.03–0.25)	0.30 (0.01–0.78)	0.22 (< 0.01–0.65)	0.34 (< 0.01–0.87)	0.12 (< 0.01–0.45)
ind^2^	0.15 (0.06–0.29)	0.21 (0.09–0.39)	0.61 (0.13–0.86)	0.43 (0.02–0.72)	0.68 (0.05–0.92)	0.23 (0.02–0.57)
*I*_A_	0%	0%	0.33% (0.01%–0.93%)	0.19% (< 0.01%–0.63%)	0.46% (< 0.01%–1.32%)	0.09% (< 0.01%–0.37%)
*V*_A_	0.08 (0.02–0.18)	0.10 (0.03–0.25)	0.31 (0.01–0.79)	0.21 (< 0.01–0.64)	0.34 (< 0.01–0.87)	0.11 (< 0.01–0.44)
*V*_PE_	0.08 (0.02–0.18)	0.10 (0.03–0.23)	0.30 (0.01–0.78)	0.22 (< 0.01–0.65)	0.34 (< 0.01–0.87)	0.12 (< 0.01–0.45)
*V*_R_	0.07 (0.02–0.18)	0.10 (0.03–0.23)	0.32 (0.08–0.78)	0.17 (< 0.01–0.61)	0.23 (0.03–0.84)	0.56 (0.24–0.82)
*V*_fixed_	0.77 (0.62–0.88)	0.69 (0.51–0.84)	0.07 (0.02–0.16)	0.40 (0.24–0.55)	0.09 (0.02–0.18)	0.21 (0.09–0.34)

Abbreviations: *h* ^2^, heritability; *I*_A_, evolvability; ind^2^, repeatability; pe^2^, permanent environmental effects; *V*_A_, additive genetic variance; *V*_fixed_, variance due to fixed effects; *V*_PE_, variance due to permanent environmental effects; *V*_R_, residual variance.

^a^ For Poisson distributed traits (start day, end day, duration) heritability values are given in the observed data state.

Body weight, hind leg length, chest girth, and ingesta‐free body fat percentage did not differ significantly between east–west and north–south migrating individuals (Bonar et al. 2022). Frequency plots of body size traits are available in the Figure S6. Heritability of body size traits ranged from 0.12 (0.01–0.57) for ingesta‐free body fat (measurements taken in March of the year of migration) to 0.27 (0.06–0.53) for hind leg length. Permanent environmental effects pe^2^ (CI) and repeatability (i.e., among‐individual differences, ind^2^ (CI)) were similarly low for ingesta‐free body fat 0.07 (0.01–0.31) and 0.19 (0.03–0.66) respectively. Permanent environmental effects for other body size traits ranged from 0.22 (0.05–0.63) for weight to 0.45 (0.17–0.84) for hind leg length. Repeatability estimates were all moderate for the remaining body size traits with 0.42 (0.14–0.86) for weight, 0.56 (0.27–0.88) for chest girth, and 0.72 (0.46–0.93) for hind leg length (Figure 1, Table 2). Proportion of variance for body size traits can be found in Figure S7, Tables S3–S6.

## 4 Discussion

Migratory behavior is a complex process that is thought to emerge from a combination of physiological, morphological, and cognitive traits (Abraham et al. 2022; Bonar et al. 2023), and research suggests that genetic loci at least partially underpin the phenotypic variation of migratory traits (see Liedvogel et al. 2011 for review and examples). Our study provides, to our knowledge, the first empirical evidence of heritability in migration behavior in ungulates. We found low heritability for broad patterns of migration timing, and moderate heritability for distance, duration, and movement rate along the migratory route. Additionally, our heritability estimates of body size traits were comparable to the current estimates of ungulate body size (e.g., Jamieson et al. 2020; Williams et al. 1994; Wilson et al. 2005). Therefore, we have confidence that our pedigree‐free approach to estimating heritability of both body size and migration behaviors has likely captured a true signal. These findings have important implications for our understanding of the evolution of migration in ungulates and for how migration behaviors may be influenced by human‐mediated environmental change.

We found moderate heritability for average movement rate during migration (h^2^ = 0.34) after accounting for age, year of migration and distance migrated. In comparison, we found low heritability of non‐migratory average movement rate (*h* ^2^ = 0.11). A genetic basis for movement traits in mule deer has been suggested by Lingle (1992) who showed that the escape gaits of mule deer, white‐tailed deer, and their F1 hybrid crosses were highly consistent. Heritability of migration behavior in ungulates has not been measured in other systems; however Gervais et al. (2020) quantified heritability of movement rate and space‐use behaviors within home ranges of roe deer ( *Capreolus capreolus* ) and found a moderate heritability (*h* ^2^ = 0.21 ± 0.08) for average daily movement speed and high heritability of distance to roads (*h* ^2^ = 0.70 ± 0.11). The genetic basis for speed and stamina has been well documented in racehorses, with genome‐wide scans identifying candidate loci correlating with speed (Kis et al. 2021; Moon et al. 2015) with heritability estimates ranging from low (*h* ^2^ range: 0.074 ± 0.012–0.124 ± 0.006; Sharman and Wilson 2023) to moderate and high (*h* ^2^ range: 0.38 ± 0.03–0.68 ± 0.05; Williamson and Beilharz 1998). The moderate heritability of movement rate suggests that the rate at which mule deer migrate has the potential to change to some degree at the population level; specifically, under rapid environmental change it may be adaptive for migrations to become faster when conditions become less predictable.

Although moderate heritability of migration timing has been found in birds (*h* ^2^ = 0.34–0.54; Moller 2001; Pulido et al. 2001), we found low heritability in migration timing in our mule deer population. Low heritability can result from the erosion of additive genetic variance by stabilizing or directional selection (Merilä and Sheldon 1999), or from increased residual variance due to variation in environment (Price and Schluter 1991). Variation due to fixed effects explained the largest proportion of phenotypic variation for migration start and end day in our models. Work with mule deer populations in the Piceance Basin by Lendrum et al. (2013) found that spring migration in mule deer differed among years and was related to the environmental variables of snow depth, and plant phenology. Similarly, Monteith et al. (2011) found the same patterns of year‐to‐year variation in migration timing, linked to southern oscillation index, snow depth and plant phenology for mule deer in the Sierra Nevada, USA. Migration timing across ungulates is known to be influenced by plant phenology (e.g., elk ( *Cervus elaphus canadensis* ) (Rickbeil et al. 2019), mule deer (Lendrum et al. 2013; Monteith et al. 2011) red deer ( *Cervus elaphus* ) (Peters et al. 2019), roe deer (Peters et al. 2019; Rivrud et al. 2016)), with species exhibiting plasticity in their migration timings in order to optimize foraging along their route. Plant phenology is tightly linked to changes in temperature (Schwarts and Reiter 2000) which contribute to yearly variation in spring green‐up.

Heritability does not necessarily imply a strong response to selection; therefore, we estimated the evolutionary potential of migration traits using the trait evolvability (Houle 1992). We measured evolvability for migration traits and found that evolvability centered on zero for migration timing and ranged from an average of 0.19% for migration duration to an average of 0.43% for migratory movement rate, meaning that migratory movement rate could change by 0.43% per generation per unit selection. While Hansen et al. (2011) found in their literature review that median evolvabilities for behavioral traits were 1.56% ± 0.39%, this range of evolvabilities for migration traits could still lead to notable responses to selection over hundreds of generations.

Estimates of individual repeatability are assumed to set the upper bound for heritability (Bell et al. 2009; Dohm 2002). Repeatability for migratory traits followed similar trends to that of heritability estimates, where repeatability of migration timing was lower than that of migration distance, duration and movement rate. The range of average repeatability across all migration traits in our study was 0.15–0.68. Bell et al. (2009) found in their meta‐analysis the average measure of repeatability of behavior was 0.37. High repeatability of migration behaviors might be linked to strong spatial fidelity exhibited by mule deer (Lendrum et al. 2013; Northrup et al. 2016; Rodgers et al. 2021). For example, Sawyer et al. (2019) found high levels (> 80%) of site fidelity in mule deer migration routes, regardless of age, reproductive status, or number of years monitored and Northrup et al. (2016), working in our system, showed high year‐to‐year fidelity in summer and winter ranges indicating they migrate to and from the same locations annually. Similarly, Mahoney and Schaefer (2002) found that the rank order of caribou ( *Rangifer tarandus* ) migration was highly consistent among individuals, and also independent of sex or age. Studies reporting consistent among‐individual differences in movement and space‐use behaviors are becoming more prevalent across taxa (Gervais et al. 2020; Hertel et al. 2019; Laforge et al. 2025; Schirmer et al. 2019), however the mechanisms underpinning individual repeatability of behaviors is still in need of exploration. We did not sample resident mule deer in this study; however Sawyer et al. (2019) sampled both migratory and resident mule deer and found that individuals did not switch strategy year‐to‐year. Sawyer et al. (2019) proposed that the high level of consistency in mule deer migration behavior is due to the strong reliance on memory for navigation. Migrations shaped largely by memory or learning through cultural transmission may be less flexible (Bracis and Mueller 2017; Jesmer et al. 2018; Merkle et al. 2019), however it is possible that migratory information may be updated or transmitted through social learning leading to a degree of flexibility in migration routes or destinations (Bartlam‐Brooks et al. 2011).

When estimating heritability using GRM‐based approaches, sample size and variance in relatedness are important factors. Small sample size can increase sampling variance, which would bias V_A_ downward, thereby decreasing heritability estimates (de Villemereuil et al. 2013). Our study and that of Gervais et al. (2020) are two examples which demonstrate a GRM‐based approach can be used to estimate heritability using samples sizes of a few hundred unrelated individuals. The GRM variance of relatedness for our study was low, because like other studies of wild ungulates (e.g., Bérénos et al. 2014; Gervais et al. 2020) our GRM was skewed toward unrelated individuals. With SNP data, the GRM variance is inversely proportional to the variance of *h* ^2^ (Visscher and Goddard 2015), however, by removing highly related individuals, we can reduce error resulting from being unable to sample the full spectrum of relatives (Zaitlen et al. 2013). Given that the relatedness of individuals in our study sample already skewed toward zero and contained very few closely related individuals our measures of heritability were mostly unchanged even after removing individuals with relatedness 0.1 or 0.05. However, population‐wide estimates using unrelated individuals can lead to an underestimation of heritability estimates compared to pedigree approaches (Vinkhuyzen et al. 2013). While these factors could bias our estimates of heritability low, we can be more confident that our higher heritability estimates are capturing truly heritable traits.

GRM‐based approaches make estimating heritability of wild populations more accessible; however, one of the challenges it presents is understanding the effect of heterogeneous landscapes on heritability estimates. Observed heritability of a trait may be partially due to related individuals sharing more similar habitats, which would result in the genetic and environmental sources of variation being potentially confounded (Kruuk and Hadfield 2007). In our system, relatives are more likely to share similar environments as offspring are philopatric to their mother. Environmental similarity also affects heritability estimates in red deer populations (Stopher et al. 2012) but did not have a substantial effect in Soay sheep ( *Ovis aries* ; (Regan et al. 2017)). In such cases, if closely related individuals are excluded from the analysis, which is the case in our study, this source of bias should be reduced because among distantly related individuals, their genomic similarity is poorly correlated with pedigree coefficients, and it is only the pedigree relationship that might be correlated with environmental similarity in this scenario (Vinkhuyzen et al. 2013).

Our study provides empirical demonstration of heritability of a migration trait. Broad patterns of migration timing have low heritability and are heavily influenced by environmental variation, while movement behavior during migration is more heritable and subject to variation in individual repeatability. Future studies should seek to quantify the heritability of more movement and space‐use behaviors that affect fitness along the migration route, such as forage tracking, and habitat selection or avoidance of anthropogenic features. Changes to the landscape resulting from human activity are resulting in the disappearance of migration routes globally (Harris et al. 2009; Wilcove and Wikelski 2008). Bonnet et al. (2022) found in their recent study that across bird and mammal populations, estimates of additive genetic variance of fitness are often substantial and more than expected; and suggests that the rates of adaptive evolution may occur faster than previously expected on a generation‐to‐generation time scale. Mule deer migrations are of interest to wildlife managers as recent anthropogenic development and climate change may threaten migratory routes (Lendrum et al. 2013; Northrup et al. 2014). Knowledge about the heritability, evolvability, and degree of plasticity of migration related traits and the evolutionary potential and speed of and speed of response to selection is crucial to make management decisions in at‐risk populations. Our findings imply that wild mule deer populations have the potential to respond to selection pressure generated global environmental changes through microevolutionary changes in migration behaviors.

## Author Contributions

**Maegwin Bonar:** conceptualization (equal), data curation (equal), formal analysis (equal), writing – original draft (equal), writing – review and editing (equal). **Eric Wootton:** data curation (equal), formal analysis (equal), writing – review and editing (equal). **Charles R. Anderson Jr.:** data curation (equal), funding acquisition (equal), resources (equal), writing – review and editing (equal). **George Wittemyer:** data curation (equal), funding acquisition (equal), resources (equal), writing – review and editing (equal). **Aaron B. A. Shafer:** conceptualization (equal), investigation (equal), project administration (equal), supervision (equal), writing – review and editing (equal). **Joseph M. Northrup:** conceptualization (equal), data curation (equal), funding acquisition (equal), investigation (equal), project administration (equal), supervision (equal), writing – review and editing (equal).

## Funding

This work was supported by the Natural Sciences and Engineering Research Council of Canada. Compute Canada.

## Conflicts of Interest

The authors declare no conflicts of interest.

## Supporting information


**Figure S1:** Principal coordinate analysis of individuals by *F*_IS_ coefficient, prior to removal based on excess heterozygosity (*n* = 155).
**Figure S2:** Principal coordinate analysis of individuals by *F*_IS_ coefficient, after removal based on excess heterozygosity (*n* = 143).
**Figure S3:** Distribution of pairwise genomic relatedness coefficients (*n* = 20,306).
**Figure S4:** Frequency distribution of log‐transformed daily average movement rate for the month of March for all 143 individuals.
**Figure S5:** Proportion of phenotypic variance of traits explained by additive genetic effects, permanent environmental effects, fixed effects and residual effects. Posterior mean and 95% CI.
**Figure S6:** Frequency distributions of body size traits for 143 individuals. Body size traits are (a) chest girth (cm); (b) hind leg length (cm); (c) ingesta‐free body fat (%); and (d) weight (kg).
**Figure S7:** Variance partitioning of body size traits. Proportion of phenotypic variance of traits explained by additive genetic effects, fixed effects and residual effects. Ches, chest girth; Leg, hind leg length; IFBF, ingesta‐free body fat percentage; Wt, weight.

**Table S1:** Summary data for unfiltered and filtered genomic relatedness matrices (GRM). The GRM was filtered based on two relatedness coefficient cut‐offs, 0.1 and 0.05.
**Table S2:** Variance component estimates and their associated ratios for body size and migration traits using the unfiltered GRM with and the GRM with 0.05 relatedness cutoff. *h* ^2^, heritability; pe^2^, permanent environmental effects; ind^2^, repeatability; *I*_A_, evolvabiltiy; *V*_A_, additive genetic variance; *V*pe, permanent environmental effects; *V*_R_, residual variance; *V*_fixed_, variance due to fixed effects.
**Table S3:** Summary of fixed and random effect for each body size animal model. All in the animal models require a measure of pairwise relatedness as a random effect, our measure of relatedness is determined using genomic relatedness matrices (GRMs). We log‐transformed response variables with continuous distributions to meet assumptions of homoscedasticity and normality of residuals.
**Table S4:** Data structure for migratory and body size traits for all individuals used in unfiltered GRM.
**Table S5:** Fixed effects for all univariate migration and body size animal models. Mean posterior values (95% CI).
**Table S6:** Narrow sense heritability (*h* ^2^) estimates (mean and 95% CI) for body size traits using the unfiltered GRM, *r* = 0.1 relatedness cutoff, and *r* = 0.05 relatedness cutoff. *h* ^2^, heritability; *V*_A_, additive genetic variance; *V*_R_, residual variance; *V*_fixed_, variance due to fixed effects.
**Table S7:** Heritability (*h* ^2^) and G‐matrix correlation values (rG) for the bivariate animal model correlating migratory movement rate and hindleg length using the unfiltered GRM and the GRM with 0.05 and 0.1 relatedness cutoff. Mean posterior (95% CI).

## Data Availability

Raw sequence reads are deposited on NCBI SRA (BioProject PRJNA850423). Associated data and analysis code has been archived on Zenodo DOI: https://doi.org/10.5281/zenodo.20736679. Supporting Information is available online.
