# Supplementary material for: The Heritability of Migration Behaviors in a Wide‐Ranging Ungulate
Source: Ecol Evol. 2026 Jul 13;16(7):e74024. doi: 10.1002/ece3.74024 (PMC13364405; doi:10.1002/ece3.74024)
Supplement: Supplementary file 1 — Figure S1: Principal coordinate analysis of individuals by F IS coefficient, prior to removal based on excess heterozygosity (n = 155). Figure S2: Principal coordinate analysis of individuals by F IS coefficient, after removal based on excess heterozygosity (n = 143). Figure S3: Distribution of pairwise genomic relatedness coefficients (n = 20,306). Figure S4: Frequency distribution of log‐transformed daily average movement rate for the month of March for all 143 individuals. Figure S5: Proportion of phenotypic variance of traits explained by additive genetic effects, permanent environmental effects, fixed effects and residual effects. Posterior mean and 95% CI. Figure S6: Frequency distributions of body size traits for 143 individuals. Body size traits are (a) chest girth (cm); (b) hind leg length (cm); (c) ingesta‐free body fat (%); and (d) weight (kg). Figure S7: Variance partitioning of body size traits. Proportion of phenotypic variance of traits explained by additive genetic effects, fixed effects and residual effects. Ches, chest girth; Leg, hind leg length; IFBF, ingesta‐free body fat percentage; Wt, weight. [file ECE3-16-e74024-s001.docx]

**The heritability of migration behaviours in a wide-ranging ungulate**

Maegwin Bonar^1*^, Eric Wootton^2^, Charles R. Anderson Jr.^3^, George Wittemyer^4^, Aaron B. A. Shafer^1^ and Joseph M. Northrup^1,5^

^1^Environmental & Life Sciences Graduate Program, Trent University, Peterborough, ON, K9L 0G2, Canada

^2^Molecular Biology & Biochemistry Undergraduate Program, Trent University, Peterborough, ON, K9L 0G2, Canada

^3^Mammals Research Section, Colorado Parks and Wildlife, Fort Collins, CO 80523, USA

^4^Department of Fish, Wildlife and Conservation Biology, Colorado State University, Fort Collins, CO, 80523, USA

^5^Wildlife Research and Monitoring Section, Ontario Ministry of Natural Resources, Peterborough, ON K9J 3C7, Canada

**Supplemental Figures and Tables:**

**Supplemental tables can be found in the file supplemental_tables.xlsx**


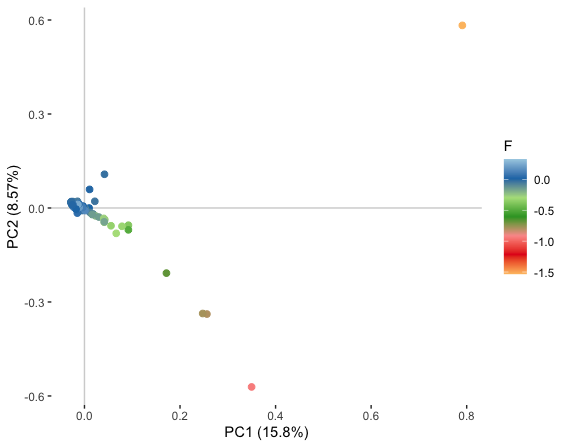


**Figure S1.** Principal coordinate analysis of individuals by F_IS_ coefficient, prior to removal based on excess heterozygosity (n = 155)


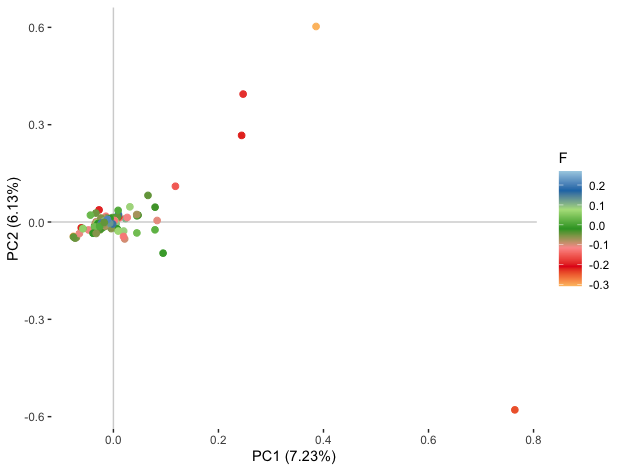


**Figure S2.** Principal coordinate analysis of individuals by F_IS_ coefficient, after removal based on excess heterozygosity (n = 143).


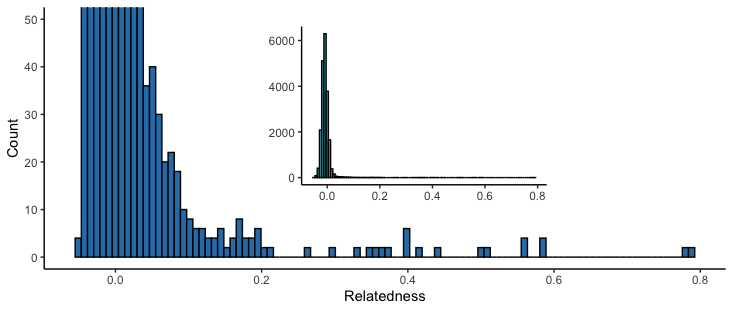


**Figure S3**. Distribution of pairwise genomic relatedness coefficients (n = 20,306)


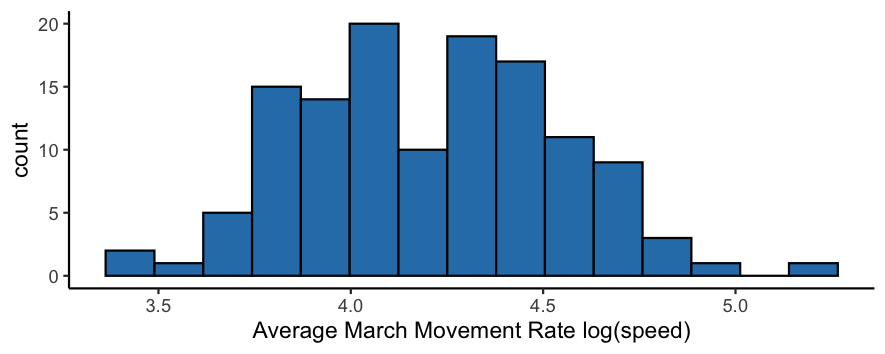


**Figure S4.** Frequency distribution of log-transformed daily average movement rate for the month of March for all 143 individuals.


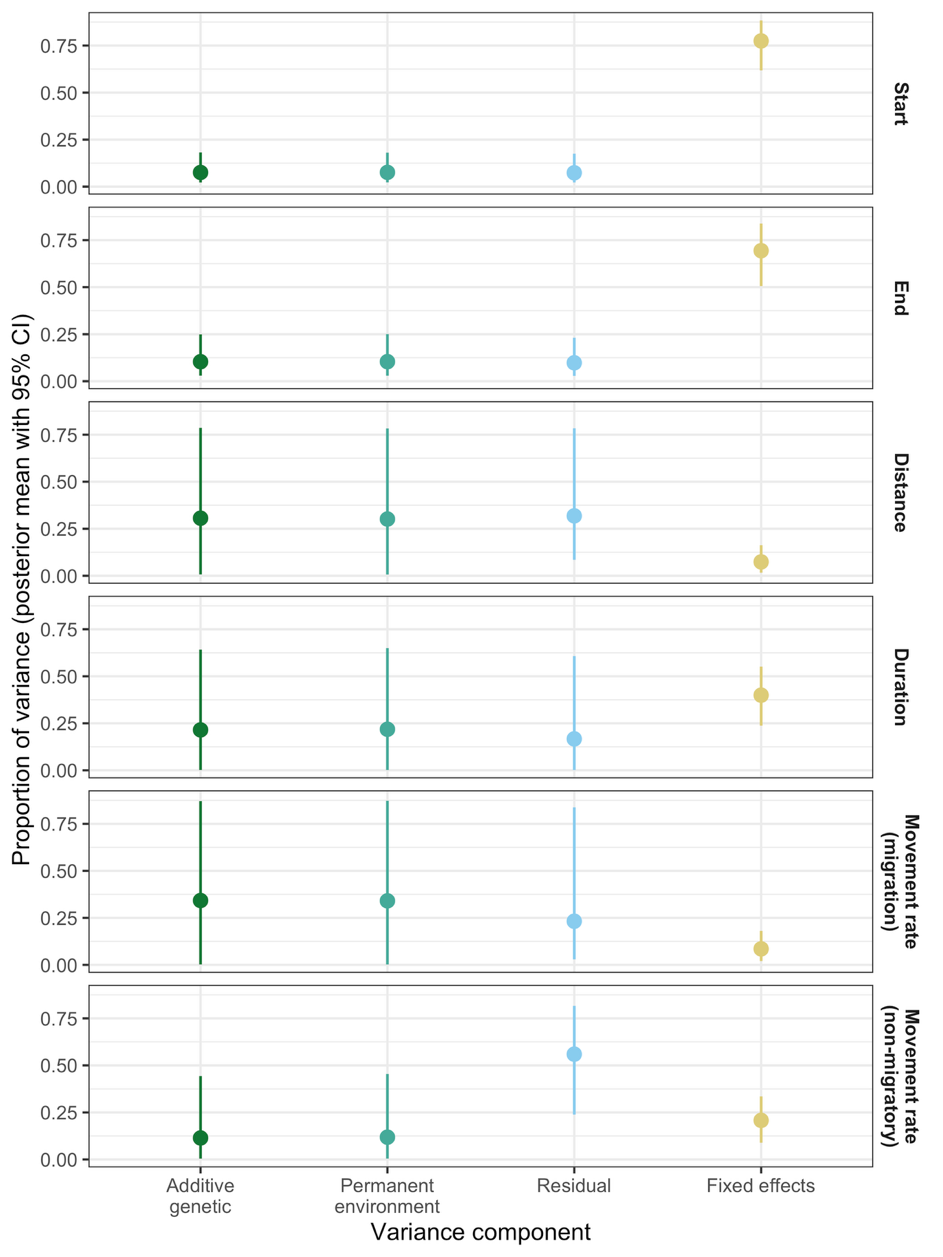


**Figure S5.** Proportion of phenotypic variance of traits explained by additive genetic effects, permanent environmental effects, fixed effects and residual effects. Posterior mean and 95% CI.


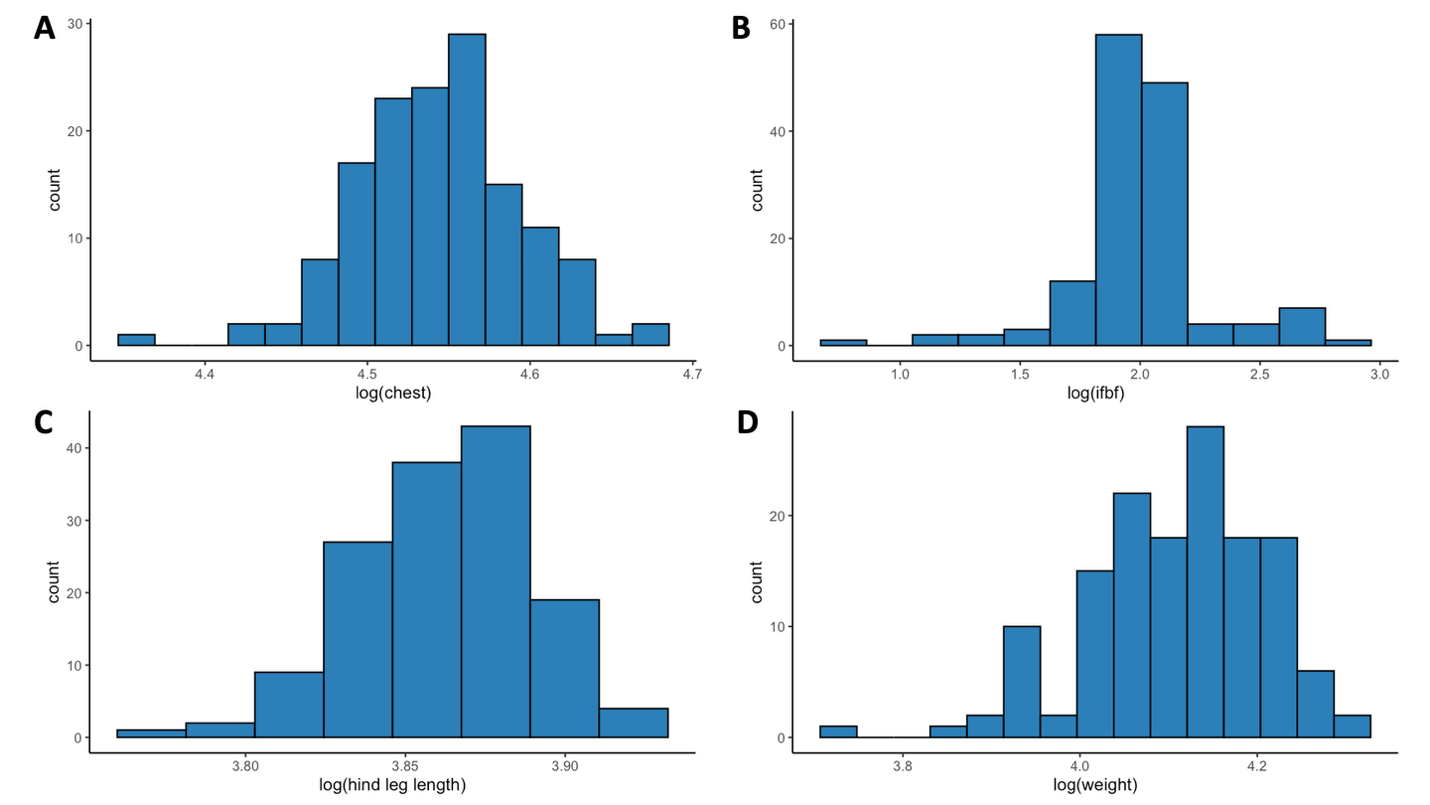


**Figure S6.** Frequency distributions of body size traits for 143 individuals. Body size traits are a) chest girth (cm); b) hind leg length (cm); c) ingesta-free body fat (%); and d) weight (kg).


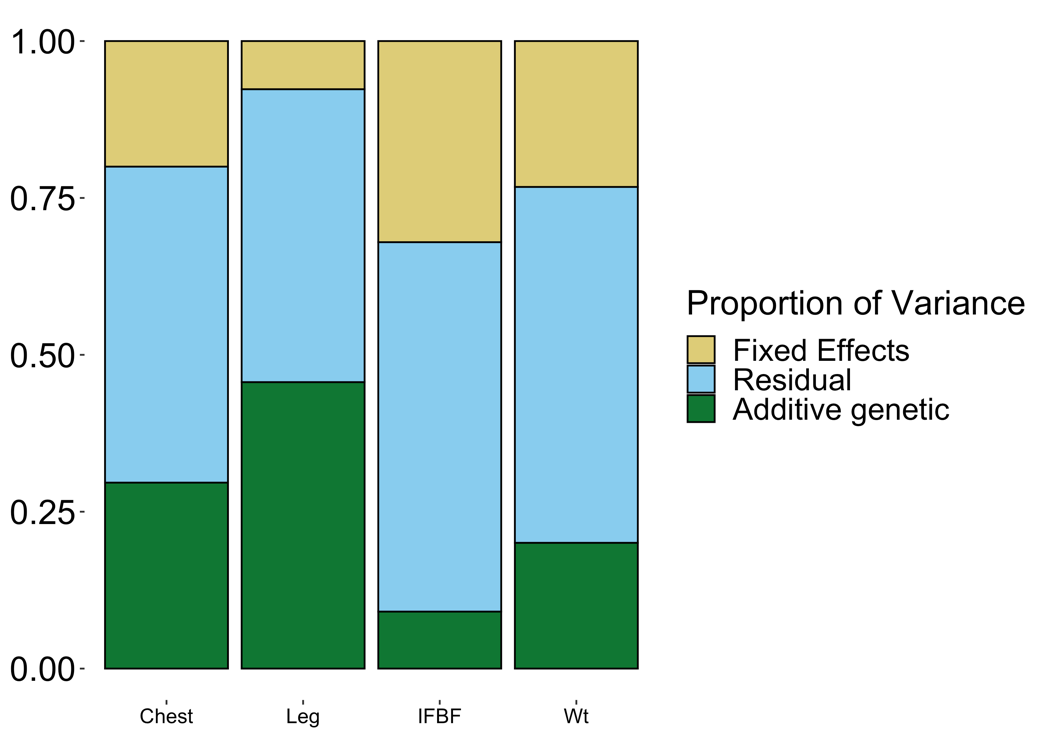


**Figure S7.** Variance partitioning of body size traits. Proportion of phenotypic variance of traits explained by additive genetic effects, fixed effects and residual effects. Ches – chest girth; Leg – hind leg length; IFBF – ingesta-free body fat percentage; Wt – weight.
